# Interaction of *Acinetobacter* sp. RIT 592 induces the production of broad-spectrum antibiotics in *Exiguobacterium* sp. RIT 594

**DOI:** 10.3389/fphar.2024.1456027

**Published:** 2024-08-01

**Authors:** Anutthaman Parthasarathy, Renata Rezende Miranda, T. J. Bedore, Lizabeth M. Watts, Pavan K. Mantravadi, Narayan H. Wong, Jonathan Chu, Joseph A. Adjei, Amisha P. Rana, Michael A. Savka, Zackery P. Bulman, Eli J. Borrego, André O. Hudson

**Affiliations:** ^1^ School of Chemistry and Biosciences, University of Bradford, Bradford, United Kingdom; ^2^ School of Chemistry and Materials Science, Rochester Institute of Technology, Rochester, NY, United States; ^3^ Thomas H. Gosnell School of Life Sciences, Rochester Institute of Technology, Rochester, NY, United States; ^4^ Agilent Technologies, Santa Clara, CA, United States; ^5^ Department of Pharmacy Practice, University of Illinois at Chicago, College of Pharmacy, University of Illinois at Chicago, Chicago, IL, United States

**Keywords:** antibiotics, AMR, MDR, bioprospecting, GNPs, antimicrobial peptides, *Exiguobacterium*, *Acinetobacter*

## Abstract

Antimicrobial resistance (AMR) is one of the most alarming global public health challenges of the 21st century. Over 3 million antimicrobial-resistant infections occur in the United States annually, with nearly 50,000 cases being fatal. Innovations in drug discovery methods and platforms are crucial to identify novel antibiotics to combat AMR. We present the isolation and characterization of potentially novel antibiotic lead compounds produced by the cross-feeding of two rhizosphere bacteria, *Acinetobacter* sp. RIT 592 and *Exiguobacterium* sp. RIT 594. We used solid-phase extraction (SPE) followed by liquid chromatography (LC) to enrich antibiotic extracts and subsequently mass spectrometry (MS) analysis of collected fractions for compound structure identification and characterization. The MS data were processed through the Global Natural Product Social Molecular Networking (GNPS) database. The supernatant from RIT 592 induced RIT 594 to produce a cocktail of antimicrobial compounds active against Gram-positive and negative bacteria. The GNPS analysis indicated compounds with known antimicrobial activity in the bioactive samples, including oligopeptides and their derivatives. This work emphasizes the utility of microbial community-based platforms to discover novel clinically relevant secondary metabolites. Future work includes further structural characterization and antibiotic activity evaluation of the individual compounds against pathogenic multidrug-resistant (MDR) bacteria.

## Introduction

Antibiotic resistance is a major threat to public health worldwide. This crisis has arisen from the abuse of antibiotics in animal husbandry and the lack of research and development of novel antibiotics (Palumbi, 2001; Ventola, 2015; Ribeiro da Cunha et al., 2019). During the golden age of antibiotics, between 1940 and 1962, their discovery and development flourished with the commercialization of 20 clinically relevant classes; however, despite the ever-growing AMR risk, only two new antibiotic classes have been developed in the last 60 years. Major pharmaceutical companies commit only limited resources towards antibiotics research, as drugs treating lifestyle diseases yield greater and more sustainable profits (Coates et al., 2002). However, due to increasing public health concerns, the field of antibiotic discovery is reviving among academic researchers, academic-industry partnerships, and small biotech companies that have delivered several antibiotics to the market over the last decade. In a recent report from the Pew Charitable Trusts, of the companies with antibiotics in clinical development, only two were large. Instead, 95% of antibiotic products developed originate from smaller firms (Trusts, 2021). In particular, the WHO released a Priority List in 2024, whereby carbapenem-resistant *Acinetobacter baumannii* and Enterobacterales resistant to carbapenem and third-generation cephalosporins were in the Critical group, and both MRSA and carbapenem-resistant *Pseudomonas aeruginosa* were in the High group (World Health Organization, 2024).

Increased efforts and novel approaches are needed as antibiotic resistance for most infectious diseases rises (Bulman et al., 2022). While some researchers have advocated for screening compound libraries, as was recently done with synthesized short macrocyclic-tethered peptides (MCPs) to identify a novel class of antibiotics targeting lipopolysaccharide transport in *A. baumannii* (Zampaloni et al., 2024), others have called for exploiting nature’s repertoire of antimicrobial products. Microbes comprise the largest gene pool available, and it is reasonable to expect they encode the machinery to synthesize a diverse source of secondary metabolites, including those with potentially novel antimicrobial activity (Scherlach and Hertweck, 2021). Among antibiotic-producing bacteria, Actinomycetes are undeniably the best-studied. However, this overrepresentation in the scientific literature has potentially limited antibiotic discovery (Smanski et al., 2016). To improve the likelihood of discovering novel and mechanistically diverse antibiotics, not only should more bacterial genera be surveyed for their biosynthesis, but the emergent properties arising in microbial communities should be explored. Members of these communities likely evolved divergent antibiotic biosynthesis and resistance phenotypes that may not manifest under axenic conditions. Additionally, some antibiotics may have signaling roles at subinhibitory concentrations, while others may act as global gene regulators (Beyersmann et al., 2017). Antibiotics could form not only the chemical basis of the interactions of the antibiotic producers with other microbes but also those with larger organisms such as plants and animals (Yim et al., 2007; Fajardo et al., 2009; Romero et al., 2011). The exploitation of new niches driven by antibiotic biosynthesis has been demonstrated in the case of bacterial symbionts of ants (Caldera and Currie, 2012).

In the past decade, bacteria from marine environments, extreme environments, caves, the deep biosphere, endophytes/epiphytes, and insect symbionts have been uncovered as promising sources of novel antibiotic compounds (Dapkevicius, 2013; Wang et al., 2014; Newman and Cragg, 2015; Martinez-Klimova et al., 2017; Matsumoto and Takahashi, 2017; Tortorella et al., 2018; Van Moll et al., 2021; Baranova et al., 2022; Pipite et al., 2022). However, microbial residents of more traditional environments can still be a rich resource for antimicrobial discovery, especially if ecological interactions are considered. Many secondary metabolites are not produced under standard laboratory monocultures, and the importance of interspecific microbial interactions in inducing otherwise cryptic biosynthetic gene clusters (BGCs) has been largely under appreciated. Nevertheless, studies have demonstrated that nearly 40%–50% of all metabolites produced in a mixed culture showed chemical novelty and antibiotic activity (Bertrand et al., 2014; Tyc et al., 2014; Tyc et al., 2017). Recognizing and responding to their fellow microbial community members via specific activation of distinct cryptic BGCs would allow bacteria to adapt to their environments appropriately (Smanski et al., 2016). Therefore, the chemical-ecological signaling between two (or more) microbes co-evolved within the same environment is expected to be stronger than microbes whose ecological ranges do not overlap.

This chemical signaling hypothesis and the chemical novelty rates reported from mixed microbial cultures motivated our search for antibiotic producers from the rhizosphere, focusing on bacteria that would naturally interact. Herein, we present the isolation and whole genome sequencing of two bacterial species, *Exiguobacterium* sp. RIT 594 and *Acinetobacter* sp. RIT 592, isolated from soil on the Rochester Institute of Technology (RIT) campus. We show that organic extracts of RIT 594 stimulated by the supernatants of RIT 592 produce antibacterial activity against type strains (ATCC) of *Escherichia coli*, *P. aeruginosa*, *Bacillus subtilis,* and *Staphylococcus aureus*, as well as known MDR clinical isolates of *S. aureus* (Diep et al., 2006; Planet, 2017), *E. coli* (Mediavilla et al., 2016), and *P. aeruginosa* (CDC, 2022). After chromatographically processing the extracts using solid-phase extraction (SPE) and liquid chromatography (LC), we isolated and identified these compounds through mass spectrometry analysis. Some of the leading candidates in our analysis are short peptides but different from MCPs.

## Results and discussion

### Bacterial isolation and antimicrobial activity screening

The two isolated strains were found to be closely associated with each other on a diversity plate, with the yellow/orange *Exiguobacterium* RIT 594 able to grow on top of the white *Acinetobacter* RIT 592 (Supplementary Figure S1). Their taxonomic identification based on whole genome sequences is shown in the Supplementary Material (Supplementary Figure S2). The genome sequence of RIT 594 was deposited in the NCBI database with the accession number QPKF00000000, and that of RIT 592 was deposited with the accession number QPKU00000000. A summary of the sequencing effort is provided in Supplementary Table S1. To simulate this interaction of the two bacteria in a controlled way, we added the sterile-filtered spent culture supernatant of RIT 592 to live cells of RIT 594, henceforth known as RIT 592/594 extract(s). In an analysis of the two monoculture extracts vs. the combined extract just described, 105 m/z features had elevated levels in the RIT 594 monoculture, 45 had elevated levels in the RIT 592 monoculture, and 46 had elevated levels only in the combined extracts (Supplementary Figure S3).

### Antibiotic activity characterization of RIT 592/594 crude bacterial extracts

Ethyl acetate extracts of spent LB culture media of *Exiguobacterium* sp. RIT 594, *Acinetobacter* sp. RIT 592 and the *Exiguobacterium* sp. RIT 594 spiked with 20% v/v of *Acinetobacter* sp. RIT 592 sterile-filtered supernatant was initially screened in disc diffusion assays, showing that the combined extract had more activity than the monoculture extracts (Supplementary Table S2). We chose 20% because a dose-response assay using a range of 5%–50% (v/v) of RIT supernatant suggested that antibiotic activity is suppressed beyond 20% (Supplementary Figure S4). Therefore, only ethyl acetate spent LB medium extracts of *Exiguobacterium* sp. RIT 594 spiked with 20% v/v of *Acinetobacter* sp. RIT 592 supernatant (henceforth called RIT 592/594 extracts) were used in our subsequent assays.

**FIGURE 1:**
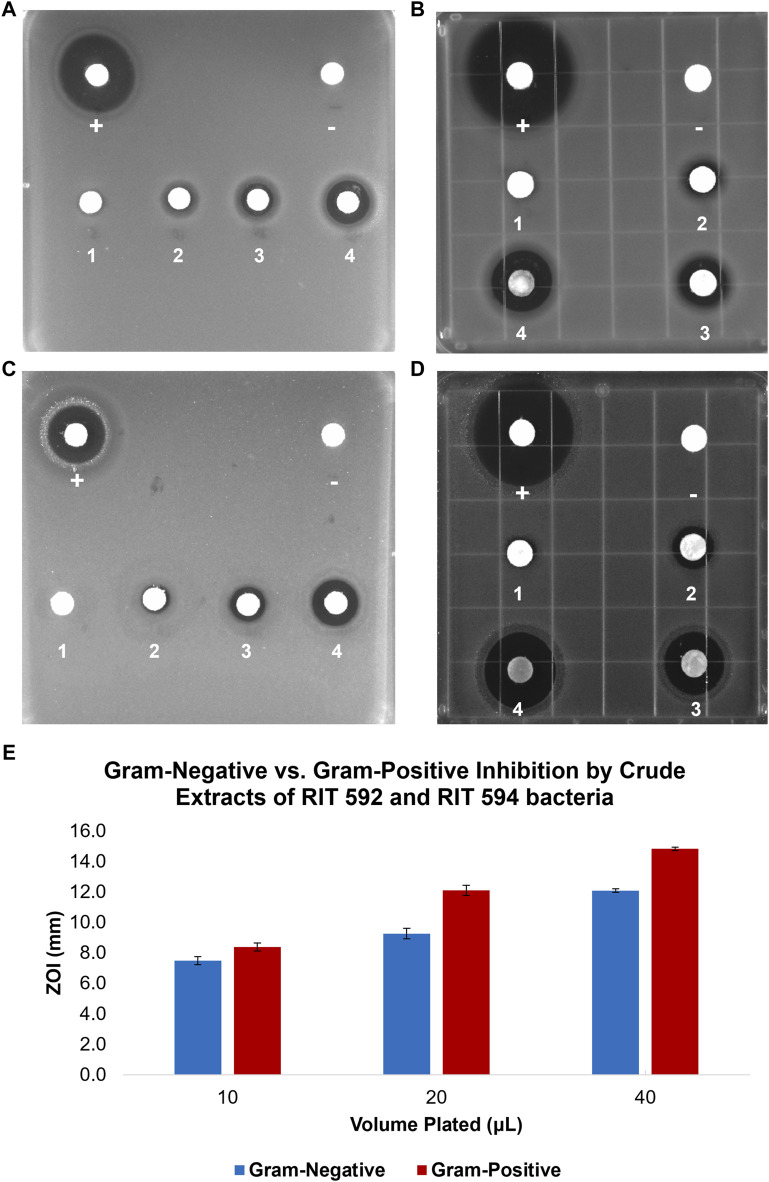
Disc-diffusion inhibitory assays of ethyl acetate spent medium crude extracts of *Exiguobacterium* sp. RIT 594 combined with 20% v/v of *Acinetobacter* sp. RIT 592 supernatant against various bacteria: *Escherichia coli* **(A)**, *Staphylococcus aureus* **(B)**, *Pseudomonas aeruginosa* **(C)**, and *Bacillus subtilis* **(D)**. Tetracycline, 0.2 mg (+, positive control); methanol, 20 μL (-, negative control); 1.25 mg (1), 2.5 mg (2), 5 mg (3), and 10 mg (4) of the crude extract. The experiments were performed in duplicates, and the average ZOI values are shown in Supplementary Table S3. **(E)** Bar graph comparing the inhibitory activities of different volumes of RIT 592/594 extracts against the Gram-negative and Gram-positive bacteria shown in panels **(A–D)**.

Disc diffusion assays were performed against four reference strains, including Gram-positive (*B. subtilis* and *S. aureus*) and Gram-negative (*E. coli* and *P. aeruginosa*) microorganisms (Figures 1A–D). Increasing amounts of RIT 592/594 crude extracts were applied to sterile paper discs equally distributed on square Petri plates inoculated with the corresponding bacterium, and the zones of inhibition (ZOIs) around each disc were measured (Supplementary Table S3). All four tested bacteria demonstrated dose-dependent susceptibility towards the RIT 592/594 crude extracts, with a moderately greater effect on the Gram-positive bacteria (Figure 1E).

### Time-kill assays of RIT 592/594 crude bacterial extracts against clinical pathogens

**FIGURE 2:**
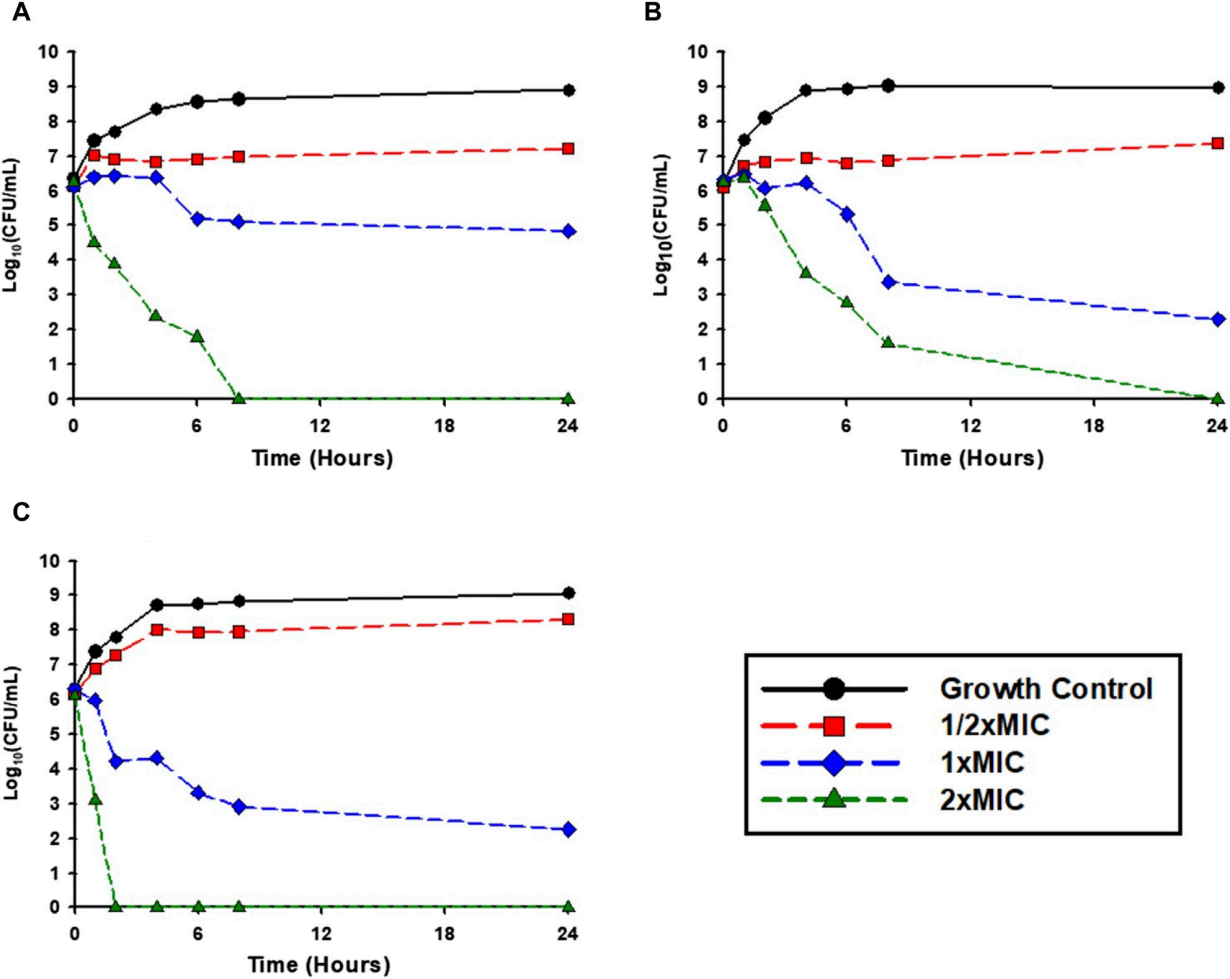
Time-kill experiments: Activity of the RIT 592/594 crude extract over 24 h in time-kill experiments. The extract was tested at concentrations of 1/2xMIC, 1xMIC, and 2xMIC against: **(A)** MRSA USA300-FPR3757, **(B)** *E. coli* MCR1_NJ, and **(C)** *Pseudomonas aeruginosa* AR-0230.

Time-kill experiments were performed as previously described (Huang et al., 2021) to assess the antimicrobial activity of the RIT 592/594 extracts against clinically relevant strains. The crude ethyl acetate extract yielded a dose-response relationship for each isolate (Figure 2). Against MRSA USA300-FPR3757, the extract failed to reduce the bacterial population at 1/2xMIC and only generated a 1.3 log_10_ CFU/mL reduction after 24 h at a concentration of 1xMIC. However, 2xMIC was bactericidal after 4 h (3.88 log_10_ CFU/mL reduction) and eradicated MRSA USA300-FPR3757 by 8 h (Figure 2A). When tested against *E. coli* MCR1_NJ, the extract was bactericidal at 1x- and 2xMIC for 24 h. *E. coli* MCR1_NJ was eradicated by the extract at 2xMIC after 24 h (Figure 2B). The extract’s bacterial killing rate was the greatest against *P. aeruginosa* AR-0230 as 2xMIC eradicated the entire inoculum by 2 h. The extract at a concentration of 1xMIC caused 3.4 and 4.0 log_10_ CFU/mL reductions at 8 and 24 h, respectively (Figure 2C).

### Isolation of the active antibiotic compounds in the RIT 592/594 extracts

**FIGURE 3:**
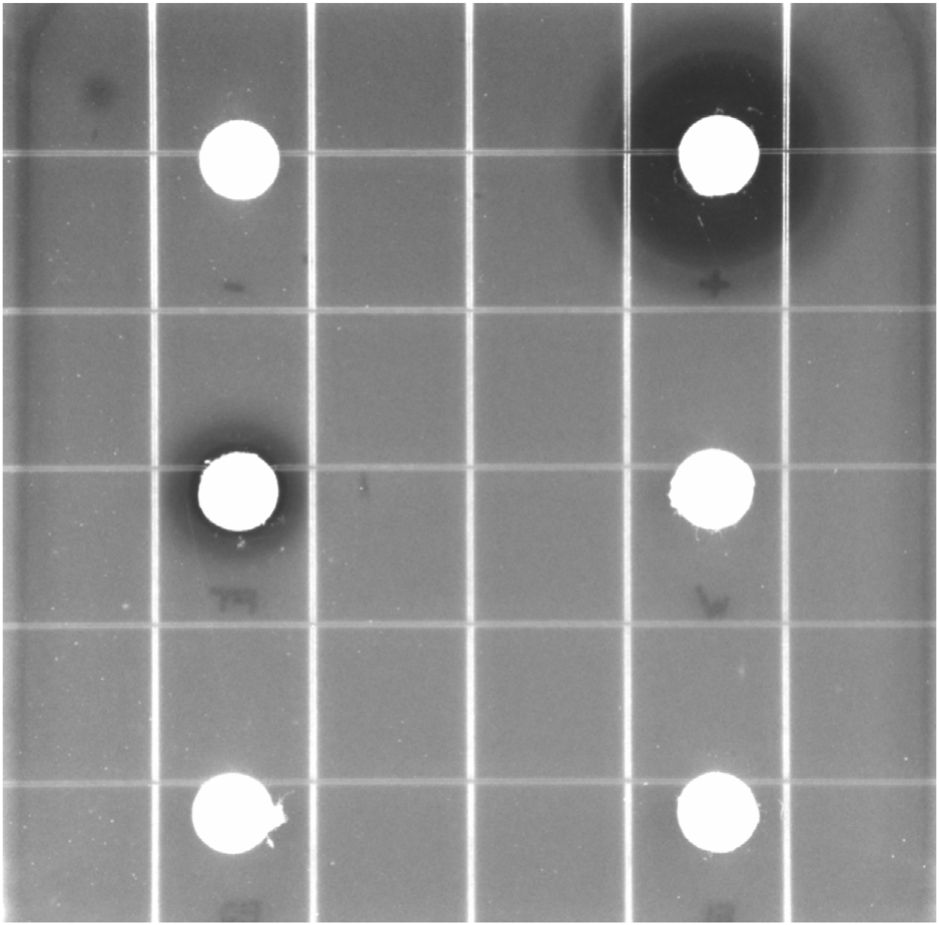
Disc-diffusion inhibitory assay against *Escherichia coli* of fractions collected after strong anion exchange (SAX) solid-phase extraction for an initial purification of the antibiotic compounds produced by *Exiguobacterium* sp. RIT 594 and *Acinetobacter* sp. RIT 592. Content of the discs: tetracycline, 0.2 mg (positive control); methanol, 20 μL (negative control); flowthrough (FT), 5 mg; wash (W), 0.25 mg; elution 1 (E1), 0.25 mg; elution 2 (E2), 0.25 mg. Zone of inhibition values (mm): tetracycline, 18 ± 2.03; methanol, 0; FT, 7.8 ± 0.64; W, 0; E1, 0; E2, 0. These data represent the mean values of three independent experiments.

The crude RIT 592/594 extracts were processed using separation methods to isolate their bioactive compounds. For the initial purification of the extracts, we used the solid-phase extraction (SPE) technique called strong anion exchange (SAX). After running the samples through the column, we tracked the antibiotic activity of all collected fractions in disc diffusion inhibitory assays against *E. coli* (Figure 3; similar procedure as mentioned above). The amounts collected in each fraction are shown in Supplementary Table S4. The flowthrough (FT) was assayed at a 250 mg/mL concentration and the other fractions at 50 mg/mL. The FT showed the highest antibiotic activity compared to the other collected fractions, with an average zone of inhibition (ZOI) value of 7.8 mm. The wash and elution fractions (E1 and E2) produced no substantial growth inhibition. Our findings indicate that SAX effectively retained part of the noninhibitory compounds produced by the two bacteria while eluting the potentially active compounds from the crude extract in the FT fraction. The lack of retention of the antibacterial active components on the SAX column suggested the presence of cationic active compounds (possibly peptides or other positively charged compounds at pH 2).

Based on these results, the SAX FT fraction was selected for further purification through high pressure liquid chromatography, and the resulting chromatogram is shown in Supplementary Figure S5. A 50 mg/mL solution of the active sample in methanol was used in the separation. A sample of a SAX FT fraction after separating a blank LB medium crude extract (no bacteria) was used as a negative control for chromatogram comparison and fraction selection for further analysis and characterization. Four peaks with UV absorption at 215 nm were prominent in the active sample compared to the blank sample due to their massive enrichment compared to the LB medium blank (Supplementary Figure S5). Three distinctive peaks (fractions B74-75, B80-81, and B84-85) were eluted within a range of 15–20%B (acetonitrile acidified with 1% formic acid) and retention times in between 10 and 26 min. Another peak with UV absorption at 215 nm was eluted at ∼51 min with a higher %B (∼85%) and was only detected in the active sample chromatogram (fraction A16-17). This suggests that these fractions likely correspond to unique compounds produced by the bacteria and were collected for subsequent analysis by mass spectrometry.

### Characterization of the antibiotic-active compounds produced by RIT 592/594

We processed the MS data through the Global Natural Product Social Molecular Networking (GNPS) database (Wang et al., 2016) to identify the potential antibiotic compounds produced by the two strains (see methods for details). Fractions collected from both active and blank samples were treated identically. Spectra from the blank samples were filtered out from those of the active samples. We chose those spectra with high abundance for further analysis (Table 1 – the complete data in the Excel file named “LibrarySearch” available as part of the Supplementary Material).

**TABLE 1:** Summary of compounds present in the LC collected fractions identified via mass spectrometry. Compounds were selected from the active samples according to abundance (>1.0 E+08) and filtered to exclude those also present in the blank fractions.

#	Fraction	MW (g/mol)	Compound structure	Common or IUPAC name	Reported activity
1	A16-17	546.62	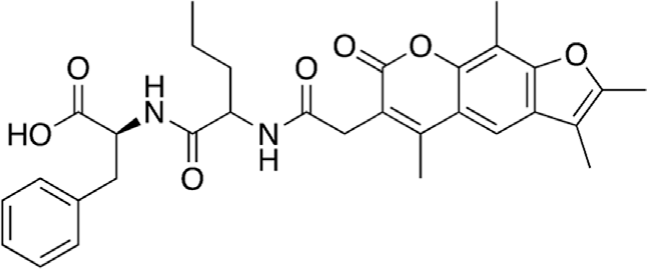	(2S)-3-phenyl-2-[2-[[2-(2,3,5,9-tetramethyl-7-oxofuro[3,2-g]chromen-6-yl)acetyl]amino]pentanoylamino]propanoic acid	-
2	A16-17	506.55	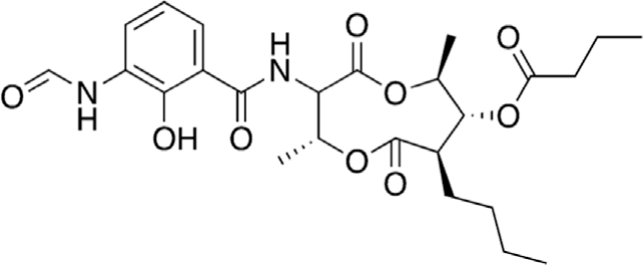	Antimycin A4	Antibiotic (fungicide) Endo and Yonehara, (1970)
3	B74-75	311.34	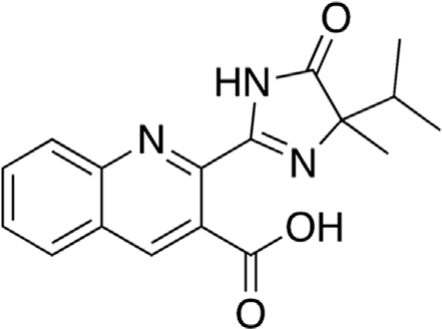	Imazaquin	Broad-spectrum herbicide (Lewis et al., 2016)
4	B74-75	260.29	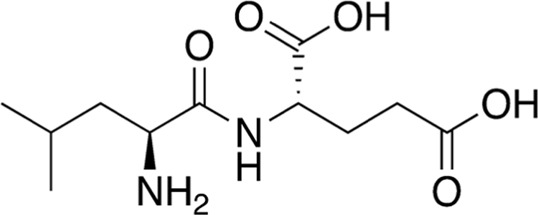	Leu-Glu	-
5	B74-75	358.48	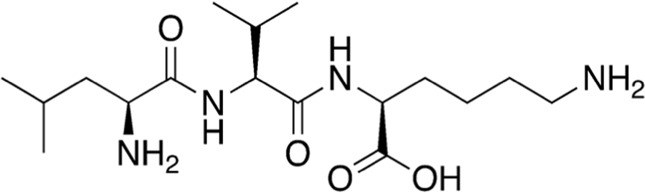	Leu-Val-Lys	-
6	B74-75	228.29	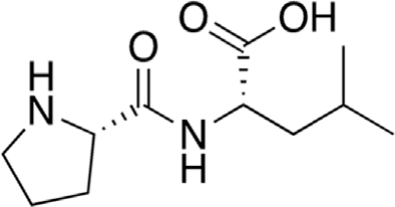	Pro-Leu	-
7	B74-75	312.37	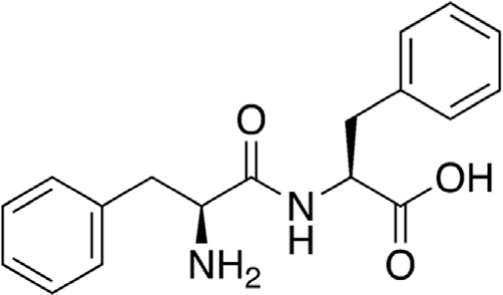	Phe-Phe	-
8	B74-75	240.26	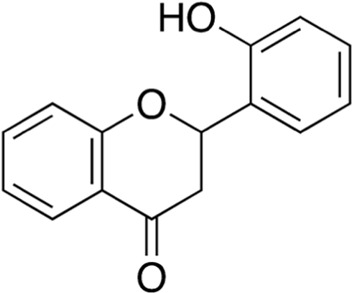	2′-Hydroxyflavanone	Anticancer, antioxidant, anti-inflammatory (Cherian et al., 2022)
9	B74-75	204.23	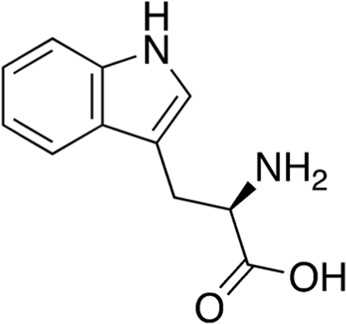	D-Tryptophan	Antibiotic adjuvant/enhancer (Bi et al., 2014)
10	B80-81	342.48	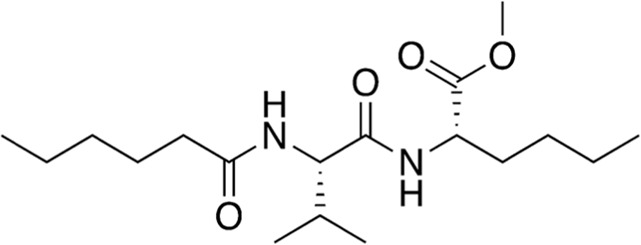	*N*-Leu-Val-hexanoate	-(Phan et al., 2022)
11	B80-81	411.47	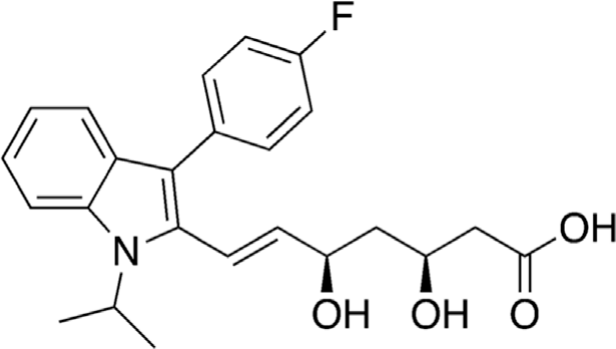	Fluvastatin	Statin (Endo, 2010)
12	B84-85	356.47	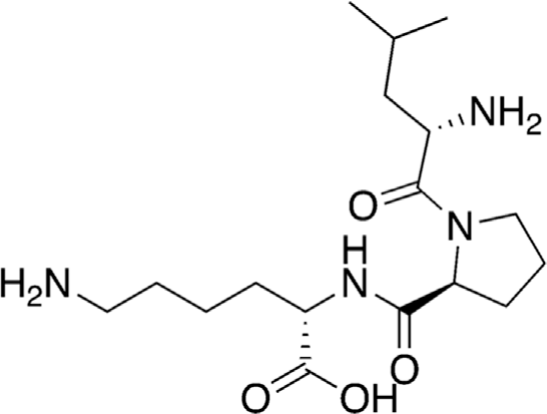	Leu-Pro-Lys	-
13	B84-85	276.29	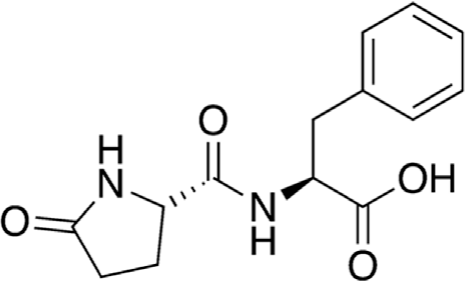	Pyroglutamyl-phenylalanine	Anti-inflammatory (Hirai et al., 2014)
14	B84-85	203.24	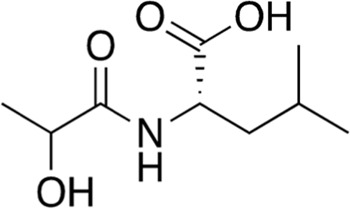	*N*-Lactoyl-Leucine	-
15	B84-85	203.24	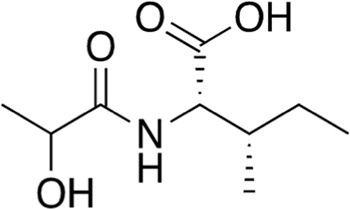	*N*-Lactoyl-Isoleucine	-

Among the most abundant compounds in our collected samples, there were various small peptides (Table 1, compounds # **4-7**, **9**, **12**), peptide derivatives (Table 1, compounds # **10**, **13-15**), and others (Table 1, compounds # **1-3**, **8**, **11**). We did a thorough literature search of each detected molecule, looking for any reported activity, and interestingly, only one molecule had antimicrobial activity against fungi (Table 1, antimycin, compound # **2**) (Endo and Yonehara, 1970). Besides this one, tryptophan (Table 1, compound # **9**) was described as an enhancer of the antibiotic activity of Trp-containing antimicrobial peptides (AMPs) due to its ability to interact with and disrupt lipid membranes (Bi et al., 2014). Other interesting compounds present in our samples had reported bioactive properties such as herbicide (Table 1, imazaquin, compound # **3**) (Lewis et al., 2016), anticancer (Table 1; 2'-Hydroxyflavanone, compound # **8**) (Cherian et al., 2022), and anti-inflammatory (Table 1; 2'-hydroxyflavanone and pyroglutamyl-phenylalanine, compounds # **8** and **13)** (Hirai et al., 2014; Cherian et al., 2022). Since these compounds with known activity do not individually have antibiotic activity, we suspect the antibiotic activity produced by the RIT 592/594 extracts could result from one or more of the other compounds detected by the MS analysis shown in Table 1. Thus, we plan to evaluate this hypothesis in our future work by either purchasing or synthesizing the compounds, testing their activity in bioassays, and confirming their biosynthesis through targeted chemical analysis.

**TABLE 2:** Unique metabolites and peptides detected in the dereplication search.

#	MW (g/mol)	Compound structure	Common or IUPAC name	Reported activity
16	679.92	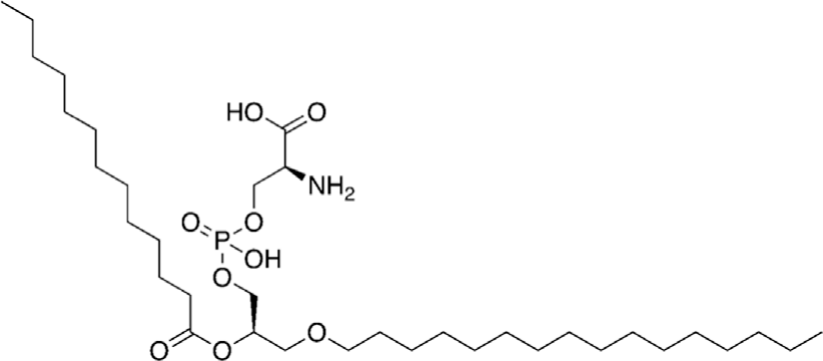	1-hexadecyl-2-tridecanoyl-glycero-3-phosphoserine	-
17	626.76	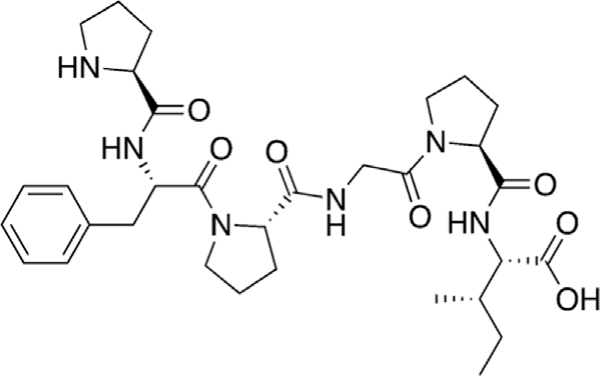	L-prolyl-L-phenylalanyl-L-prolylglycyl-L-prolyl-L-isoleucine	Cathepsin B inhibitor (Lee and Lee, 2000)
18	866.07	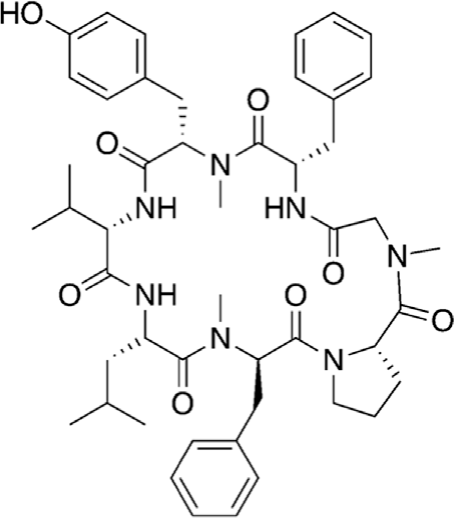	Cordyheptapeptide C	Cytotoxic (Chen et al., 2012; Klein et al., 2021)
19	1141.30	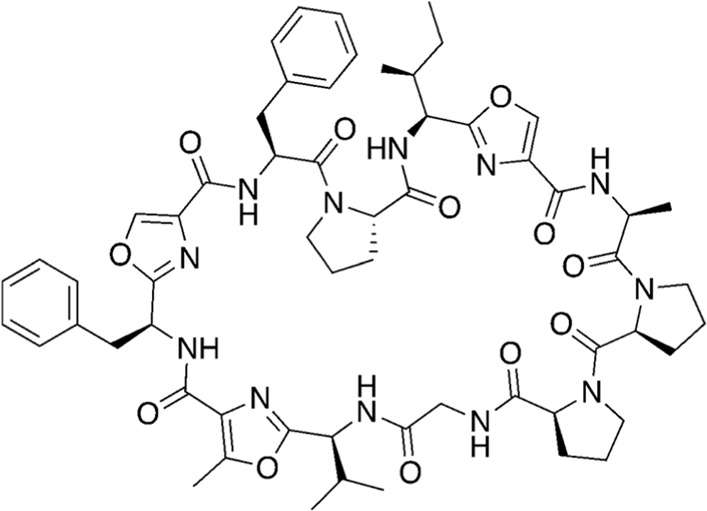	Wewakazole	Cytotoxic (Nogle et al., 2003; Lopez et al., 2016)
20	1465.80	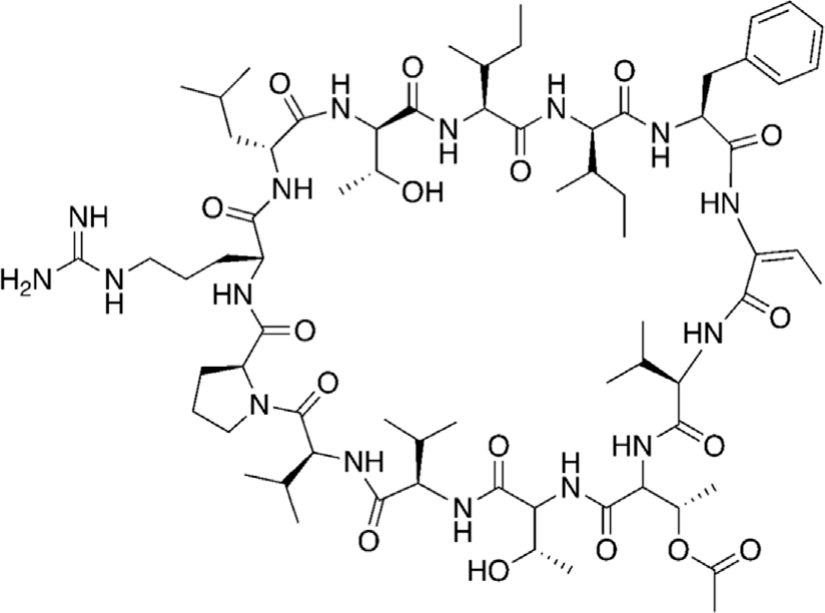	Tolybyssidin A	Antibiotic (antifungal) Jaki et al., 2001; Kar et al., 2022

Additionally, we processed our data using dereplication tools (Mohimani et al., 2017; Gurevich et al., 2018; Mohimani et al., 2018) to identify potentially unique peptidic natural products (PNPs) and/or metabolites present in our samples (see methods for details). These dereplication tools can search a spectral database to identify known PNPs and/or guide the discovery of new ones, even if the reference spectra are not present among the existing libraries (Mohimani et al., 2017). The results from this search are shown in Table 2. The dereplication tools found one metabolite (Table 2, compound # 16) and four peptides (Table 2, compounds # 17-20). All peptides had reported biological activities, including cathepsin B inhibition (compound # 17) (Lee and Lee, 2000), cytotoxicity (compounds # 18 and 19) (Nogle et al., 2003; Chen et al., 2012; Lopez et al., 2016; Klein et al., 2021) and, interestingly, antimicrobial (compound # 20) (Jaki et al., 2001; Kar et al., 2022).

### Predictions of BGCs for antibiotic biosynthesis using antiSMASH

Genome mining was performed using two different iterations of the antiSMASH program (Blin et al., 2019; Blin et al., 2023). Strict settings in either version 5.0 or 7.0 returned no “hits” except for the terpene/carotenoid BGCs for RIT 594. AntiSMASH 5.0 suggested two possible BGCs for terpenes in RIT 594 (Supplementary Figure S6), as well as other BGCs such as betalactone, arylpolyene, rudimentary sactipeptide, and betalactone-NRPS for RIT 592 under the “relaxed” setting (Supplementary Figure S7). Indeed, in the light of the current study, two BGCs from RIT 592 (Supplementary Figure S7) seem interesting. Region 10 contains an arylpolyene BGC with 20% of the genes similar to the berninamycin BGC. Berninamycin is a cyclic thiopeptide first isolated from *Streptococcus bernensis* (Abe et al., 1988; Lau and Rinehart, 1994). Region 22 contains a possible hybrid non-ribosomal peptide synthesis-beta-lactone BGC with the closest known BGC belonging to fengycin (similarity 13%). Fengycin is a cyclic lipopeptide antifungal produced by *B. subtilis* F-29-3 (Vanittanakom et al., 1986; Sur et al., 2018). In both the BGCs under discussion, at least half of the genes have functions annotated, while the other half have unknown functions (likely due to their unusual sequences).

AntiSMASH 7.0 suggested only the arylpolyene terpene/carotenoid BGCs under the “relaxed” setting. Under the “loose” setting (which can cause false positives), however, it showed BGCs responsible for the biosynthesis of oryzanaphtopyrans, dipeptide aldehydes, an S-layer glycan, several undefined saccharides, and a saccharide-carotenoid for RIT 594, plus two saccharide BGCs (one of which was a predicted polyketide BGC with 4% similarity to the xantholipin BGC) for RIT 592. Within the putative oryzanapthopyran BGC, only three biosynthetic genes were recognized, but six transport related genes were predicted. In all cases, there was low sequence similarity of the predicted BGCs to known BGCs, except for the carotenoid/terpene BGCs (>30%). We were able to demonstrate changes in the pigmentation of RIT 594 from orange to yellow or brownish (Supplementary Figure S8) under the conditions used to generate the combined RIT 592/594 extracts, suggesting modification of terpenes by RIT 594 in response to RIT 592 supernatants. It is pertinent that RIT 594 has two identified terpenoid/carotenoid type BGCs (Supplementary Figure S6). Comparing the results for antiSMASH versions, we can surmise that older versions could offer more promise to unravel BGCs with insufficient homology to known examples (which could represent novel variants).

Interestingly, the oryzanaphthopyrans are aromatic polyketides with quinone moieties found in the rare actinobacterium *Streptacidophilus oryzae* (Chen et al., 2022). Earlier studies of an antibiotic producing *Exiguobacterium mexicanum* strain by other researchers have reported a dihydroergotamine type trione active molecule (Shanthakumar et al., 2015). Antibacterial activity is also reported for pigments from the *Exiguobacterium* genus (Mekala et al., 2020). Tedesco and colleagues reported that their antioxidant and nematocidal compound producing *Exiguobacterium* sp. Strain KRL4 contains unknown phytoene synthases and a hitherto unknown siderophore pathway (Tedesco et al., 2021), whereby nearly half of all the proteins annotated had unknown functions. This underlines the difficulty of predicting bioactive compound types purely from *in silico* analysis of the genomes of bacteria from unusual genera, where often the BGCs may not be very similar to well-known antibiotic BGCs. Connecting identified active compounds to their cognate BGCs is challenging, particularly if shorter peptides are the major bioactive compounds identified, like in the present case, where bioinformatics analysis did not unambiguously identify BGCs corresponding to 3 types of cyclic peptides (Table 2). Nonetheless, the possibility of cyclic peptide biosynthesis in RIT 594/592 is not ruled out. It may be simply that most of the concerned proteins do not have well-known homologs detected by bioinformatics programs like antiSMASH.

## Conclusion

Cross-feeding the two strains, *Exiguobacterium* RIT 594 and *Acinetobacter* RIT 592, yields antibiotic extracts with activity against MDR strains. The most abundant compounds in our collected samples were bioactive compounds with acidic chemical properties and included small peptides and peptide derivatives, among others, as well as cyclic peptides that were identified by dereplicating tools. Nonetheless, predictions based on genome mining so far offer some tantalizing possibilities but fall short of definitive identification, and OMIC approaches may be required to identify the corresponding BGCs. The mechanism of cross-feeding leading to induction of the antibiotic synthesis is still unclear. However, a few possibilities can be tested since the application of size exclusion concentrators in the supernatant preparation would be able to identify if a protein component or a smaller peptide/organic molecule is responsible which would provide the foundation for more detailed studies via proteomics/metabolomics. Future work will include further structural characterization and antibiotic activity evaluation of the individual compounds against a more comprehensive list of pathogenic multidrug-resistant bacteria.

## Materials and methods

### Media, chemicals, bacterial strains, and consumables

Bacterial culture media were bought from Merck, agar and agarose from Fluka, primers from Invitrogen, and sterile filters (0.4 and 0.2 μm) from Corning Inc. All other chemicals were bought from Merck, USA, and Sigma-Aldrich, USA, unless mentioned otherwise. *Escherichia coli* ATCC 25922 and *P. aeruginosa* ATCC 27853 were derived from the ATCC type strains sub-cultured and maintained by Dr. Savka. Square and round Petri plates of different dimensions were purchased from VWR, Corning, and Fisher Scientific. Chromatography solvents were obtained from Fisher Chemical (Ottawa, Canada).

### Isolation of bacterial strains

Soil samples were collected from various sites around the Rochester Institute of Technology (RIT), NY, USA, with the help of a hand shovel, washed with 70% ethanol, and stored in sterile 50 mL Falcon tubes. 1 g of soil was suspended in 5 mL of sterile LB medium and grown overnight at 30°C with shaking at 200 rpm. The cultures were spun at 3000 × g for 5 min on a tabletop centrifuge to sediment the soil particles. Clear culture free of soil particles was pipetted out and serially diluted (10^–1^ to 10^–10^) plated on LB agar plates, and grown overnight at 30°C. After visual observation of the “diversity” plates, selected colonies were picked and re-streaked on fresh LB plates and grown overnight at 30°C. The re-streaking process was repeated until pure cultures were obtained. The strains reported in this study originate from a white oak tree rhizosphere soil sample on the RIT campus.

### 16S rRNA gene analysis

Colony PCR was performed using the primers 341F (CCTACGGGNGGCWGCAG) and 805R (GACTACHVGGGTATCTAATCC) for the V3-V4 region (Klindworth et al., 2013) using the GoTaq Green Mix containing the GreenTaq polymerase from Genescript Inc. PCR conditions were denaturation at 95°C for 5 min, followed by 30 cycles at 94°C for 1 min, 50°C for 1 min (annealing) and 72°C for 1 min, with a final extension at 72°C for 10 min. The amplified products were visualized by electrophoresis on a 1% agarose gel pre-stained with ethidium bromide in a model 250 electrophoresis chamber (Life Technologies, USA), with the 1 kb Plus DNA ladder (Invitrogen) as a standard at 70 V for 45 min and 1× TAE as the running buffer. The amplified products were purified using a PCR cleanup kit (Bio Basic Canada Inc.), quantified with a Nanodrop One microvolume UV-visible spectrophotometer (Thermo Scientific) and Sanger-sequenced with 341F as the sequencing primer at the Genewiz sequencing facility, NJ, United States. The sequences generated were compared using the Basic Local Alignment Search Tool (blastN) (Altschul et al., 1990) to identify the genera using the default parameters.

### Bacterial growth

*Exiguobacterium* sp. RIT 594 (Parthasarathy et al., 2022) and *Acinetobacter* sp. RIT 592 colonies were grown separately in starter cultures of 5 mL of Luria-Bertani (LB) broth (2 x RIT 592 and 3 x RIT 594) shaking at 150 rpm, 37°C, for ∼17 h. Each liquid culture was scaled-up to 100 mL and grown shaking at 100 rpm, 30°C, for ∼17 h. Next, the RIT 592 cultures were centrifuged at 5,000 rpm, room temperature (RT), for 20 min, and the resulting supernatants were poured onto the combined 300 mL of RIT 594 whole cultures. The combined 500 mL of the bacterial cross-fed culture (RIT 592/594) was poured into another 500 mL of sterile LB and grown shaking at 100 rpm, 30°C, for ∼48 h. This process was repeated until 10 L of liquid co-culture was produced.

### Ethyl acetate extraction of RIT 592 and RIT 594 co-culture medium

Scaled-up liquid co-cultures of *Exiguobacterium* sp. RIT 594 and *Acinetobacter* sp. RIT 592 was centrifuged at 6,000 rpm for 20 min at 4°C, and the supernatant was separated from the cellular pellet. Sodium chloride was added to the supernatant, and the solution was acidified with HCl to pH ∼2. Liquid-liquid organic extractions were performed with 250 mL of ethyl acetate per 1 L of media. The extracted organic layers were combined, dried with anhydrous sodium sulfate, filtered, and concentrated using a rotary evaporator (BUCHI, USA). The resulting extract crude was resuspended in methanol, and the samples were dried using a SpeedVac (Eppendorf, USA). A total of 0.725 g of crude extract was produced from 10 L of *Exiguobacterium* sp. RIT 594 and *Acinetobacter* sp. RIT 592 liquid co-cultures. Blank extractions were also performed using uninoculated LB medium to serve as controls.

### Time-kill experiments against clinical pathogens

Crude extracts of spent media from RIT 594 (with RIT 592 stimulation) were also tested for their ability to kill clinically relevant bacterial isolates in time-kill experiments as previously described (Bulman et al., 2017). The multidrug-resistant isolates used in time-kill experiments were (relevant resistance genes listed after each isolate): MRSA USA300-FPR3757 (*mecA*) (Diep et al., 2006), *E. coli* MCR1_NJ (*mcr-1, bla*_NDM-5_ *, strA, strB, aac*(*6′*)*-Ib-cr, bla*_OXA-1_ *, arr-3, sul1, sul2, tet*(*A*)) (Mediavilla et al., 2016) and *P. aeruginosa* AR-0230 (*aac(3)-Id*, *aadA2*, *dfrB5*, *bla*_OXA-4,_ *bla*_OXA-50_, *tet*(*G*), *bla*_VIM-2_) (CDC, 2022). Time-kill experiments were conducted in cation-adjusted Mueller Hinton broth and a starting bacterial inoculum of ∼10^6^ cfu/mL for each isolate. The minimum inhibitory concentration (MIC) for the crude extract against all 3 clinical isolates, determined using broth micro-dilution according to the Clinical and Laboratory Standards Institute (CLSI) Guidelines, was 6.25x (equivalent to metabolites extracted from 6.25 mL of spent media) (CLSI, 2015). Crude extract concentrations were tested at 1/2xMIC, 1xMIC, and 2xMIC in time-kill experiments, and viable colony counts were obtained after 0, 1, 2, 4, 6, 8, and 24 h. Bactericidal activity was defined as viable bacterial reductions of ≥3log_10_ CFU/mL from baseline.

### Solid-phase extraction of antimicrobial molecules from crude bacterial extracts

Solid-phase extraction (SPE) of the antimicrobial compounds was performed using Strata^®^ strong anion exchange (SAX) columns (55 µm particle size; 70 Å pore size; 500 mg sorbent mass; 6 mL total volume capacity) (Phenomenex, USA), and an SPE 12-port vacuum manifold (Agela Technologies, USA). The samples (acidified crude ethyl acetate extracts from either the bacteria or blank LB controls) were dissolved in 6 mL of a 10% methanol/water (v/v) solution to load in the columns (150 mg extract/column).

*Separation protocol for SAX columns:* 1) columns were conditioned with 6 mL of methanol; 2) columns were equilibrated with 6 mL of nanopure water; 3) columns were loaded with 6 mL of bacterial crude extracts prepared as previously described (FT); 4) columns were washed with 6 mL of nanopure water (W); 5) the first elution was done with 6 mL of 70% methanol/water (v/v) (E1); 6) the second elution was done with 6 mL of 2% formic acid in methanol (v/v) (E2). Steps 3-6 fractions were collected, dried in a SpeedVac (Eppendorf, USA), and stored at −20°C until further use.

### Disc-diffusion inhibitory assays

The reference bacterial strains *E. coli* ATCC 25922 (Minogue et al., 2014), *S. aureus* ATCC 25923 (Treangen et al., 2014), *P. aeruginosa* ATCC 27853 (Cao et al., 2017), and *B. subtilis* BGSC 168 (Zeigler et al., 2008), used in previous studies in our lab (Cavanaugh et al., 2021), were grown in 5 mL liquid LB shaking at 37°C, 150 rpm, for 16 h. Each culture was then centrifuged at 6,000 rpm, RT, for 20 min, and the cell pellet was resuspended in 5 mL of autoclaved phosphate-buffered saline (PBS, pH 7.4) to obtain an inoculum with OD_600_ of 0.1. Molten agar (40 mL) was inoculated with 400 µL of the reference strain PBS suspension and poured into a square Petri dish. Blank sterile paper discs (6 mm, BD Biosciences, USA) were placed onto the agar surface and distributed evenly spaced throughout the plate. For antibiotic activity evaluation of the bacteria extracts, the crude was diluted in methanol to a concentration of 250 mg/mL and loaded onto the discs in increasing dosages: 1.25, 2.5, 5, and 10 mg. To evaluate the SPE chromatography fractions, the FT was dissolved in methanol at a 250 mg/mL concentration and all other fractions at 50 mg/mL. Additionally, tetracycline (0.2 mg from a 10 mg/mL solution) and methanol (20 μL) were used as positive and negative controls, respectively. The plates were incubated at 37°C, for 17 h, and imaged using Chemidoc MP (Bio-Rad, USA) in the colorimetric settings. The zones of inhibition (mm) around each disc were measured using the ImageJ software (NIH, USA). These assays were performed in duplicates and were analyzed using Microsoft Excel (Microsoft, USA).

### Liquid chromatography of SAX FT fractions

Separations of the antimicrobial active FT fractions from SAX/SPE were performed on the NGC Quest 10 Chromatography System equipped with a BioFrac fraction collector (Bio-Rad, USA) and a reverse-phase Zorbax Eclipse XDB-C18 semi-preparative column (9.4 × 250 mm, 5 μm, Agilent Technologies, USA). The samples (SAX FT crude from either the bacteria or blank LB) were dissolved in methanol at a concentration of 50 mg/mL. Method parameters: manual injection with an injection volume of 200 μL; mobile phase composed of purified water (A) and 0.1% formic acid in acetonitrile (v/v, B); flow rate of 2.5 mL/min; fraction size of 3 mL; total run time of 53 min. Program: 0% B – 3 min, 0%–15% B – 5 min (gradient), 15% B – 5 min, 15%–20% B – 10 min (gradient), 20% B – 15 min, 20%–100% B – 15 min (gradient), 100% B – 2 min, 100%–0% B – 5 min (gradient). The resulting chromatograms were analyzed using the ChromLab software provided by the instrument’s manufacturer. Compared to the blank sample chromatogram, the fractions corresponding to UV peaks present only in the active sample chromatogram were selected for further analysis. The chosen fractions were freeze-dried and stored at - 20°C until further use.

### Mass spectrometry

Samples were reconstituted in 1 mL of 50% acetonitrile with 0.1% formic acid and transferred to an autosampler vial. Direct injection was performed by injecting 50 µL using a Dionex Ultimate 3000 connected to a Q Exactive Plus mass spectrometer (Thermo Fisher, USA) without any column using a flow rate of 100 μL/min. Solvent A was 0.1% formic acid in water (v/v), and solvent B was 0.1% formic acid in acetonitrile (v/v), each at 50% during the 3 min method. Molecules were ionized using a HESI source in the positive mode. Full scans were performed with a resolution of 70,000 at m/z 200, with an AGC target of 1e6 and a maximum injection time of 240 ms over a scan range of 180–2700 m/z.

### Mass spectrometry data analysis

Compound identities were analyzed through the Global Natural Products Social Molecular Networking (GNPS) (Wang et al., 2016). LC-MS/MS vendor data files were converted into the. mzML format via GNPS for subsequent analysis. Spectral libraries were searched with MOLECULAR-LIBRARYSEARCH-V2 (version release_28) using the search analog option and minimum peak intensity of 10. Molecular networking was performed with METABOLOMICS-SNETS-V2 (version release_30) using the search analog option and minimum peak intensity of 10. Spectra from blanks were filtered out before networking analysis. Spectra for known natural products were explored with DEREPLICATOR (version 1.2.8) (Mohimani et al., 2017) with the VarQuest search analog option (Gurevich et al., 2018) and DEREPLICATOR_PLUS (version 1.0.0) (Mohimani et al., 2018). Except where indicated, all other parameters for GNPS tools were set as default.

## Data Availability

The genome sequence data presented in this study are deposited in the National Library of Medicine/NCBI repository with the accession numbers QPKU00000000 (Acinetobacter RIT 592) and QPKF00000000 (*Exiguobacterium* RIT 594).
