# Supplementary material for: Interaction of Acinetobacter sp. RIT 592 induces the production of broad-spectrum antibiotics in Exiguobacterium sp. RIT 594
Source: Front Pharmacol. 2024 Aug 1;15:1456027. doi: 10.3389/fphar.2024.1456027 (PMC11324575; doi:10.3389/fphar.2024.1456027)
Supplement: Supplementary file 3 [file DataSheet1.docx]

**Supporting Information**


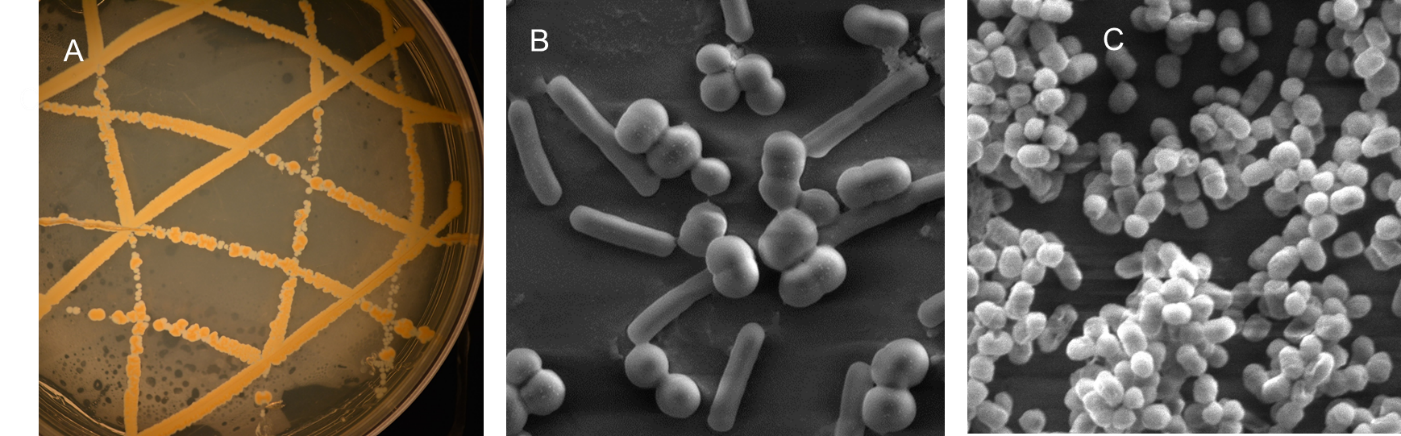


**Supporting Figure 1**. A) Petri plate (LB) of a soil dilution series showing larger yellow colonies of the RIT 594 strain growing over smaller white colonies of the RIT 592 strain. B) and C) are scanning electron micrographs of a mixed culture containing cocci of RIT 594 and ca. 2 μm rods of RIT 592 at a magnification of 9800 ×, and pure cultures of RIT 594 (magnified 16000 ×), respectively. Note the close physical contact between the two strains and the doublet/triplet morphology of the cocci in the mixed culture in B).


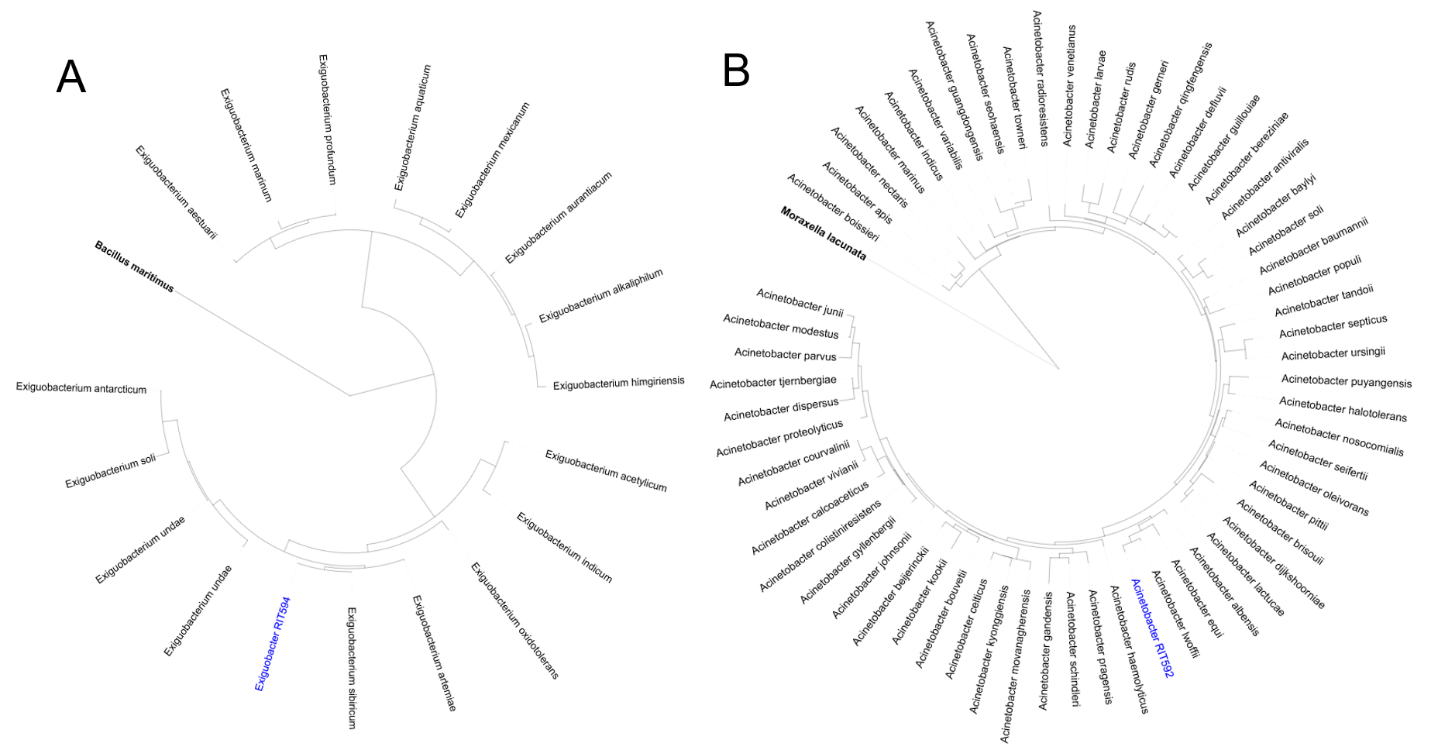


**Supporting Figure 2.** Rooted phylogenetic trees based on the 16S rRNA gene for A) *Exiguobacterium* sp. RIT 594 (blue); the nearest relative is *E. sibiricum*. *Bacillus maritimus* (bold) is taken as an out-group; tree scale = 0.001. B) *Acinetobacter* sp. RIT 592 (blue); the nearest relative is *A. lwoffii*. *Moraxella lacunata* (bold) is taken as an out-group; tree scale = 0.1.

**Supporting Figure 3.** High-resolution LC-MS analysis of ethyl acetate extracts. A) HPLC-MS traces of extracts of RIT 594, RIT 592, and the stimulated sample RIT 594 containing 20% RIT 592 supernatants; Column = Agilent Eclipse XDB-C18 column (3.5 µm, 2.1 × 150 mm), flow rate = 0.3 ml/min, mobile phase A = 0.1% formic acid in water and B = acetonitrile, respectively. Program: hold at 90% B 1 min, gradient 5-95% B 21 min, hold at 95% B 3 min, gradient 90:10% B 1 min. The mass analysis was obtained using an Agilent 6545 Q-TOF mass spectrometer with ESI capillary voltage +3.5 kV. Mass data (from m/z 70-1200) were collected using Agilent MassHunter Acquisition software (v. B.06) MS/MS was performed in a data-dependent acquisition mode. B) Venn-diagram showing the informatics analysis of the 195 m/z features whose levels are elevated. 104 are unique to RIT 594 and more abundant in the RIT 594 monoculture compared to the combined extract, while 45 are unique to RIT 592 and more abundant in the RIT 592 monoculture than the combined extract. 46 had increased levels in the combined extract compared to both monocultures.

**Supporting Figure 4.** Dose response of *E. coli* to extracts derived from RIT 592 cell-free supernatants filtered into live cultures of RIT 594, using disc diffusion assay. Discs 1, 2, 3, 5 and 6 contained 12.5 mg of the corresponding extract; the volume % of the RIT 592 supernatant added to the RIT 594 culture in each case was as follows: disc 1 = 5%; disc 2 = 10%; disc 3 = 20%, disc 6 = 40% and disc 5 = 50%. Disc 4 contained 0.2 mg of tetracycline as a positive control.

**
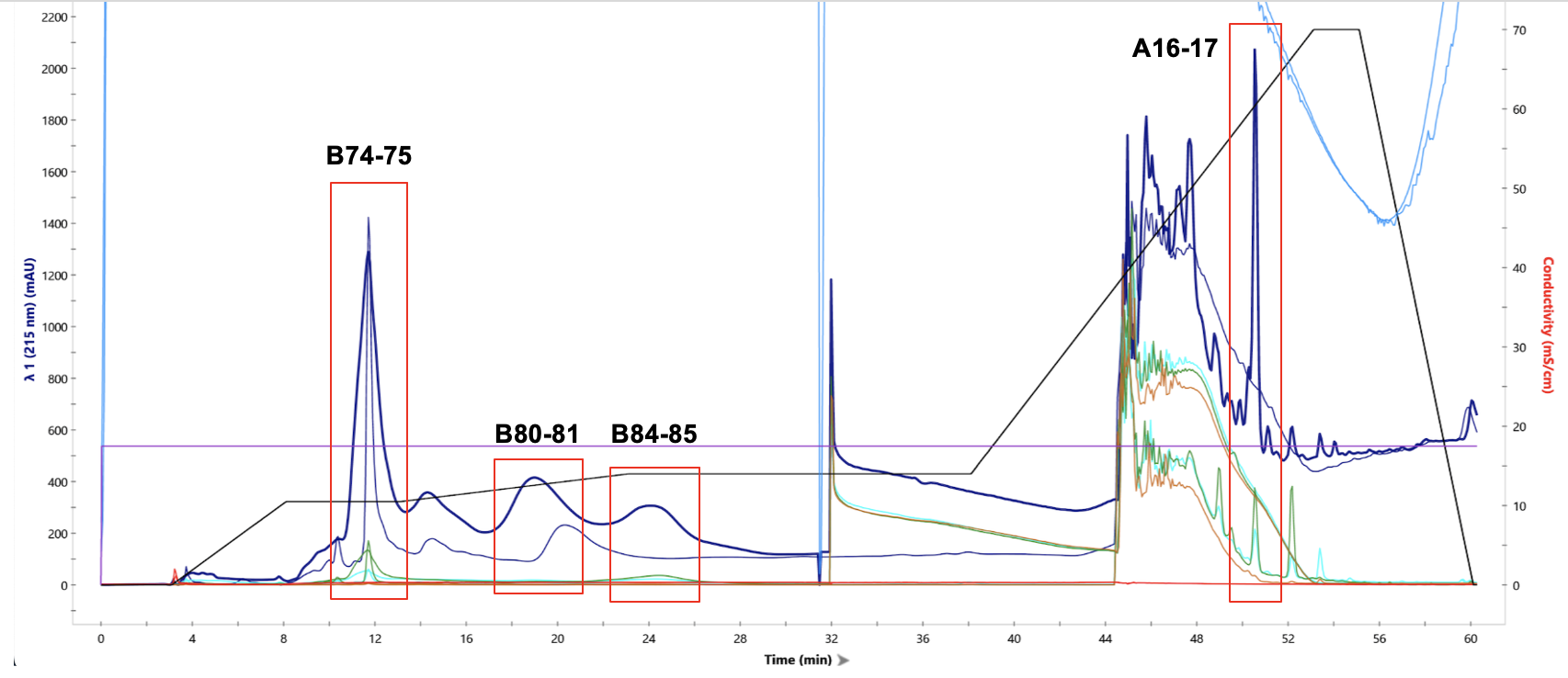
**

**Supporting Figure 5.** Semi-preparative C18 reverse-phase liquid chromatography was performed with the SAX FT fraction to further purify and isolate the bioactive compounds produced by *Exiguobacterium* sp. RIT 594 and *Acinetobacter* sp. RIT 592. Samples were injected as 50 mg/mL solutions in methanol. The chromatogram of the active sample was overlayed with that of a blank sample (SAX FT fraction from a blank LB medium – no bacteria). The bolded traces correspond to the active sample chromatogram and the nonbolded to the blank. The red squares indicate the fractions that were collected for analysis from both active and blank samples. The traces are λ1 = 215 nm (navy), λ2 = 255 nm (teal), λ3 = 280 nm (green), λ4 = 495 nm (brown), conductivity (red), % B (black), system pressure (blue), and flow rate (violet).


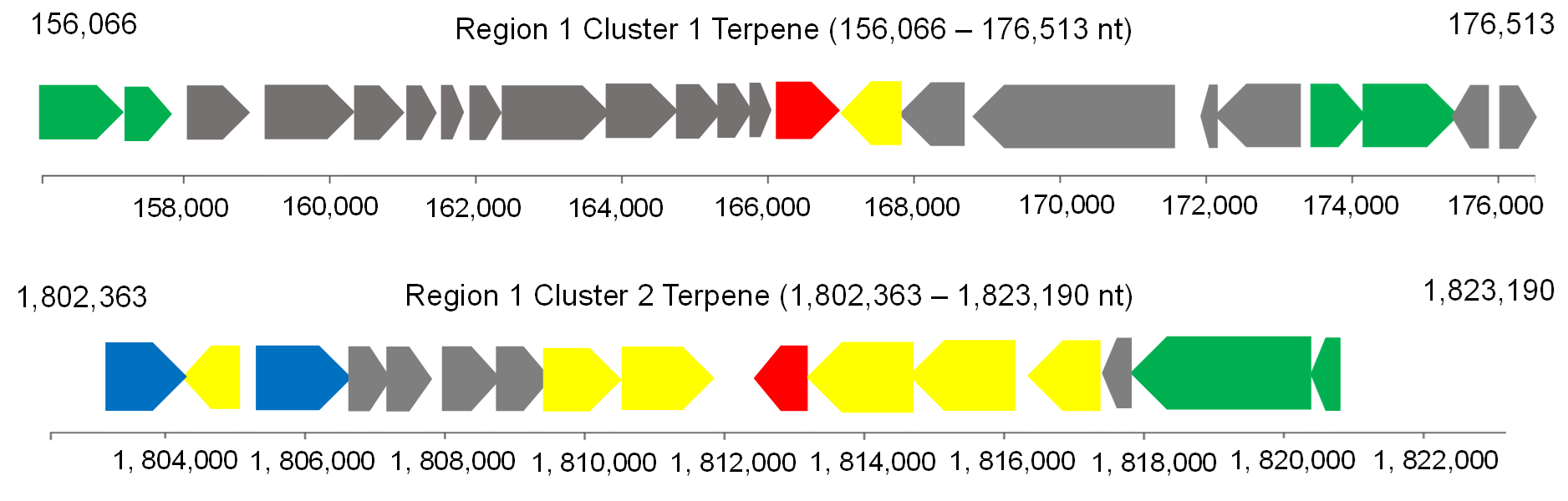


**Supporting Figure 6.** antiSMASH 5.0 analysis of the RIT 594 genome with the “relaxed” setting. Region 1 contains two BGCs encoding terpene biosynthesis with the coordinates shown. Cluster 2 is identical to a BGC known from other *Exiguobacterium* sequencing projects. Green = regulatory genes; Blue = transport-related genes; Red = core biosynthetic genes; Yellow = additional biosynthetic genes; Grey = other genes.


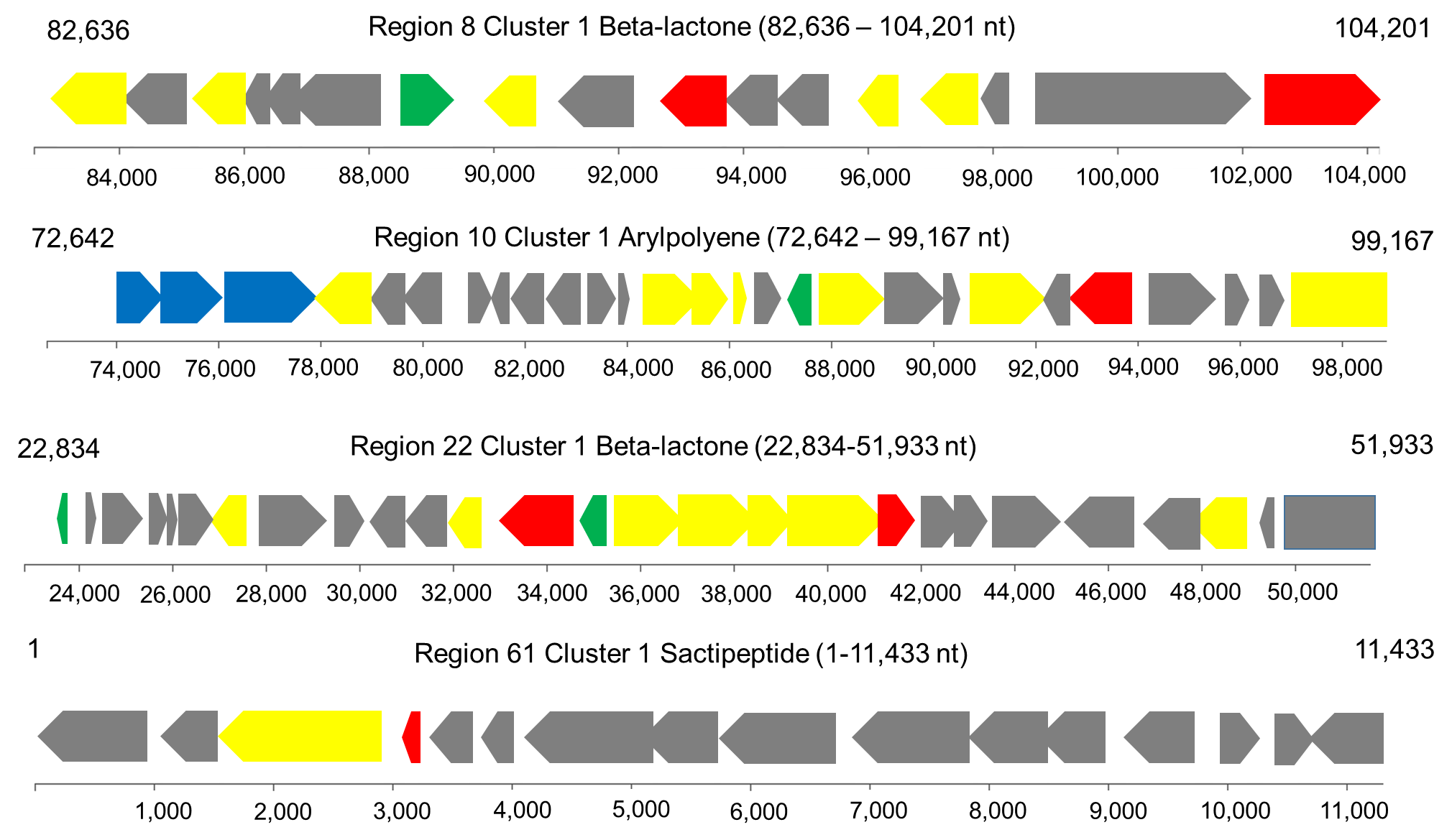


**Supporting Figure 7.** antiSMASH 5.0 analysis of the RIT 592 genome with the “relaxed” setting. Regions 8 and 22 contain beta-lactone BGCs. Region 10 contains an arylpolyene BGC with 20% of the genes similar to the berninamycin BGC, while region 22 contains a possible hybrid non-ribosomal peptide synthesis-beta-lactone BGC with the closest known BGC belonging to fengycin (similarity 13%). Green = regulatory genes; Blue = transport-related genes; Red = core biosynthetic genes; Yellow = additional biosynthetic genes; Grey = other genes.


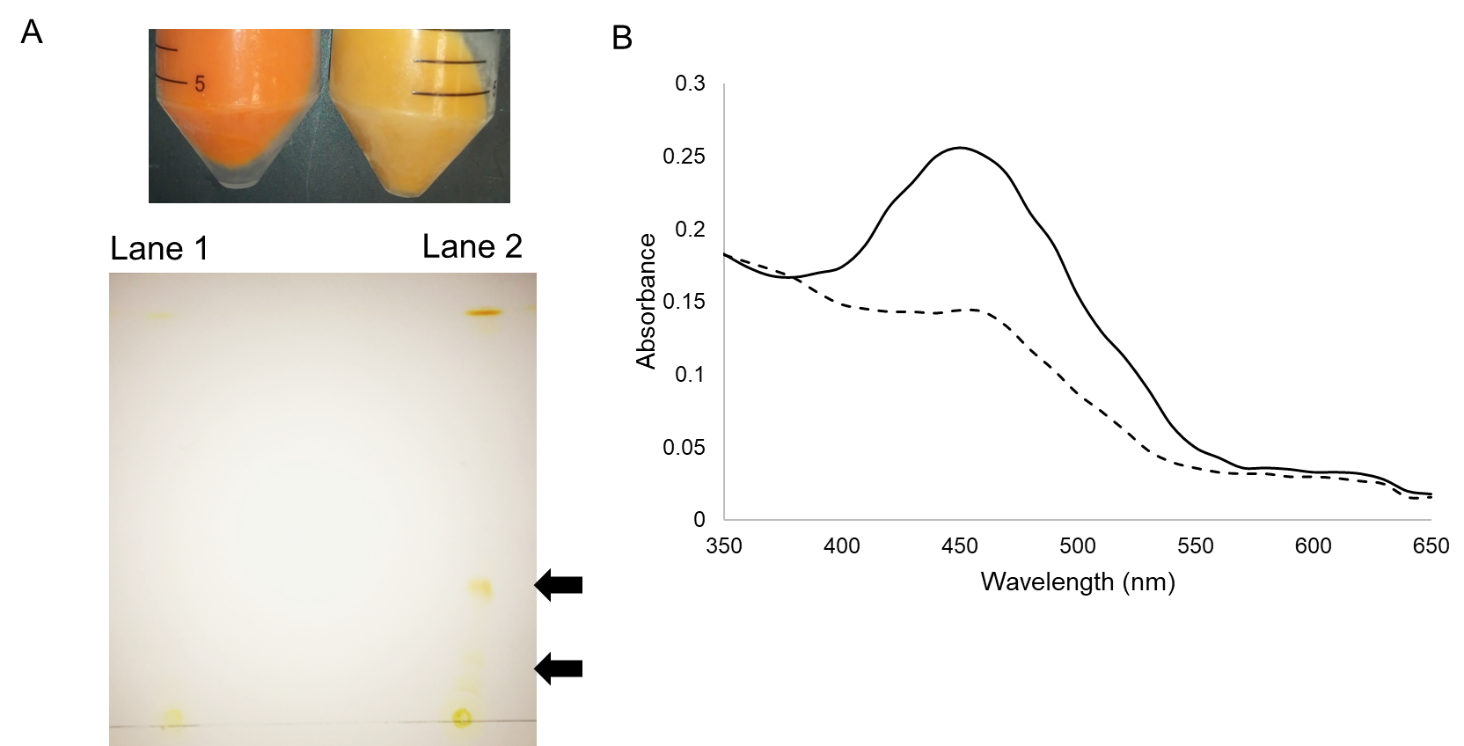


**Supporting Figure 8.** A) Difference between pigment extracts when RIT 594 is grown alone (orange cells, lane 1) vs grown in the presence of supernatants of RIT 592 (yellow cells, lane 2) for 48 h. The pigments were visualized by reverse phase thin layer chromatography on silica gel 60 RP-18 F_245_S with a 60:1 mixture of dichloromethane and methanol as the mobile phase. B) The extracts from A) differ in their visible absorption spectra in the 450-550 nm region; the broken line represents the spectrum corresponding to lane 1 and the continuous line indicates the spectrum corresponding to lane 2. The additional spots in Lane 2 indicated by black arrows in A, correlate to the spectral changes from 450-550 nm.

**Supporting Table 1**. Genome sequencing summary for strain RIT 592 and RIT 594

| Organism | Accession no. | Genome size (bp) | %GC content | Genome Coverage (X) | No. of contigs | No. of ORFs | No. of tRNAs | No. of rRNAs |
| --- | --- | --- | --- | --- | --- | --- | --- | --- |
| *Exiguobacterium* sp. RIT 594 | QPKF00000000 | 3,089,569 | 47.24 | 161 | 24 | 3208 | 62 | 3 |
| *Acinetobacter* sp. RIT 592 | QPKU00000000 | 6,069,814 | 36.52 | 68 | 1062 | 6087 | 42 | 6 |

**Supporting Table 2.** Comparison of zone of inhibition (ZOI) values from disc-diffusion inhibitory assays of ethyl acetate spent medium crude extracts of *Exiguobacterium* sp. RIT 594, *Acinetobacter* sp. RIT 592 and the combined 592/594 extracts against various reference bacteria. The values shown are averages of three independent trials using 25 mg each.

|  |  | ***E. coli***  **ATCC 25922** | ***S. aureus***  **ATCC 25923** | ***P. aeruginosa***  **ATCC 27853** | ***B. subtilis***  **BGSC 168** |
| --- | --- | --- | --- | --- | --- |
| **Sample** | **Amount** | **ZOI (mm)** | | | |
| **Tetracycline** | 0.2 mg | 22 ± 0.2 | 26 ± 0.6 | 16 ± 0.5 | 24 ± 0.1 |
| **Methanol** | 20 μL | 0.0 | 0.0 | 0.0 | 0.0 |
| **Crude Extract RIT 594** | 25 mg | 10 ± 0.7 | 12 ± 0.2 | 10.2 ± 0.5 | 13 ± 0.1 |
| **Crude Extract RIT**  **592** | 25 mg | 13 | 10.3 ± 0.9 | 9.9 ± 0.3 | 10.5 ± 0.4 |
| **Crude Extract RIT 592/594** | 25 mg | 21 ± 0.5 | 24 ± 0.4 | 20 ± 0.6 | 26.3 ± 0.8 |

**Supporting Table 3.** Zone of inhibition (ZOI) values from disc-diffusion inhibitory assays of ethyl acetate spent medium crude extracts of *Exiguobacterium* sp. RIT 594 combined with 20% v/v of *Acinetobacter* sp. RIT 592 supernatant against various bacteria.

|  |  | ***E. coli***  **ATCC 25922** | ***S. aureus***  **ATCC 25923** | ***P. aeruginosa***  **ATCC 27853** | ***B. subtilis***  **BGSC 168** |
| --- | --- | --- | --- | --- | --- |
| **Sample** | **Amount** | **ZOI (mm)** | | | |
| **Tetracycline** | 0.2 mg | 21.0 ± 0.141 | 25.6 ± 2.970 | 15.8 ± 0.849 | 23.4 ± 0.071 |
| **Methanol** | 20 μL | 0.0 | 0.0 | 0.0 | 0.0 |
| **Crude Extract 1** | 1.25 mg/5 μL | 0.0 | 0.0 | 0.0 | 0.0 |
| **Crude Extract 2** | 2.5 mg/10 μL | 7.2 ± 0.247 | 7.9 ± 0.778 | 7.8 ± 0.495 | 8.9 ± 0.990 |
| **Crude Extract 3** | 5 mg/20 μL | 8.9 ± 0.106 | 10.9 ± 0.424 | 9.6 ± 0.707 | 13.3 ± 0.849 |
| **Crude Extract 4** | 10 mg/40 μL | 12.0 ± 0.106 | 14.1 ± 0.354 | 12.2 ± 0.566 | 15.6 ± 1.414 |

*The data represent the mean values of two independent experiments.

**Supporting Table 4.** Solid-phase extraction yields per collected fraction of an average separation with strong anion exchange (SAX).

| **SAX** | |
| --- | --- |
| **Fraction** | **Amount (mg)** |
| **FT** | 61 |
| **Wash** | 10 |
| **E1** | 2 |
| **E2** | 10 |

*Ethyl acetate spent medium extracts were dissolved in 10% methanol to load into the columns. The above yields correspond to 106 mg of loaded crude extracts from two separate runs of SAX. FT = flowthrough; E1-2 = SAX elution fractions 1-2.

**Supporting Methods**

**Imaging of petri plates and chromatograms**

Petri plates (color pictures) were illuminated using a Leica Quebec Darkfield Colony Counter CCT-001 and photographed using a Nikon DX AF-S Nikkor 18-55 mm camera. An MP Chemidoc Imaging System (Bio-Rad) was used to record black and white images using the manual colorimetry settings and an exposure time of 0.3 s. All images were exported to tiff or jpeg formats and further processed using the Microsoft PowerPoint program.

**Genomic DNA isolation**

Genomic DNA was isolated from strains using 5 ml pure cultures in LB medium grown overnight at 30°C, using the GenElute bacterial genomic DNA isolation kit (Sigma-Aldrich, USA) according to the manufacturer’s protocol, and quantified using the Nanodrop One spectrophotometer.

**Genome sequencing and assembly**

Using the Nextera XT library prep kit (Illumina) and Nextera XT index kit (Illumina), 1 ng genomic DNA from each bacterial strain was processed to construct indexed libraries, following the manufacturer's protocol. The DNA concentration of each library was quantified using the DNA HS protocol on Qubit 3.0, and the average fragment size of each library was measured using the DNA 1000 chip kit on the Agilent 2100 Bioanalyzer. Each library was then manually normalized to a concentration of 4 nM in molecular-grade water, and 5 µL from each 4 nM library was pooled. The mixture of pooled, 4 nM libraries was denatured and diluted to a loading concentration of 12 pM, following the manufacturer's protocol. Finally, the denatured and diluted 6 pM pooled library mixture was sequenced using the MiSeq Reagent Kit v3 on the Illumina MiSeq for 2 × 151 cycles at the Rochester Institute of Technology Genomics Facility. Adapter trimming was performed automatically on the Illumina MiSeq during FASTQ generation. Trimmed reads were assembled de novo with Unicycler version 0.3.0b. FASTA genome assemblies were filtered using the Filter sequences by length tool on Galaxy Version 1.1 to remove sequences shorter than 200 bp, prior to uploading to Genbank.

**Strain identification and phylogenetic trees**

The strain identification based on primers for the V3-V4 region of the 16S rRNA gene was confirmed by using the whole 16S rRNA gene sequence derived from the genome. The sequences were fed into the Interactive Tree of Life (ITOL) server v5 (<https://itol.embl.de/>) (Letunic and Bork 2021) to generate phylogenetic trees.

**Predictions of secondary metabolite production**

The whole genomes of the strains RIT 592 and RIT 594 were fed into the antiSMASH servers 5.0 and 7.0 (Blin et al. 2019; Blin et al. 2023), with all the default detection options enabled to detect potential gene clusters involved in secondary metabolite biosynthesis. The strictness criterion was set at relaxed to enable the discovery of clusters encoding less characterized metabolites.

**Scanning electron microscopy (SEM)**

Samples for SEM were prepared by the following procedure. The polymer pieces covered with biofilm were rinsed gently with the buffer to remove non-adherent cells. The films were soaked in 2% glutaraldehyde in phosphate buffered saline (PBS) buffer pH 7.4 for fixation of cells for 2 h at room temperature. The samples were rinsed 3 times using the same buffer used for the fixative for 5 minutes each rinse. The samples were dehydrated in 50–80% graded ethanol for 10 min each (steps of 50%, 70% and 80%), followed by rinsing 10 min with 95% ethanol twice and finally 3 rinses of 100% ethanol from a newly opened bottle for 15 min each. All the liquid was removed by pipetting and the samples were stored at 4°C overnight sealed with Parafilm. Prior to SEM the samples were coated for two min with gold-palladium using an SPI sputter coater to mitigate charging in the electron beam. The SEM was performed at a voltage of 5 kV using a Mira3 Tescan field-emission SEM at the Rochester Institute of Technology (RIT) Nanoimaging Lab.

**Global metabolomic comparison via HPLC-MS analysis**

Separations were performed on an Agilent 1290 Infinity II system (Palo Alto, CA), with a mobile phase flow rate of 0.3 mL/min. The metabolites were assayed using an Agilent Eclipse XDB-C18 column (3.5 µm, 2.1 × 150 mm), where the mobile phase A and B were 0.1% formic acid in doubled distilled water and acetonitrile, respectively. Initial conditions were 90:10 A:B, held for 1 minute, followed by a linear gradient to 5:95 at 21 min, then held at 5:95 until 24 min. Column re-equilibration was performed by returning to 90:10 A:B at 25 minutes and holding until 32 minutes. The mass analysis was obtained using an Agilent 6545 Q-TOF mass spectrometer with ESI capillary voltage +3.5 kV, nitrogen gas temperature 325°C, drying gas flow rate 8.0 L/min, nebulizer gas pressure 30 psig, fragmentor voltage 130 V, skimmer 45 V, and OCT RF 750 V. Mass data (from m/z 70-1200) were collected using Agilent MassHunter Acquisition software (v. B.06). Mass accuracy was improved by infusing Agilent Reference Mass Correction Solution (G1969-85001). MS/MS was performed in a data-dependent acquisition mode.

**Data analysis of global metabolome samples**

Peak deconvolution and integration were performed using Agilent ProFinder (v. B.06). Bioinformatics were performed using Agilent’s Mass Profile Professional (v. 13.1). Chromatographic peaks were aligned across all samples. Peak areas were normalized by converting to log2 and applying a 75% percentile shift. Significance analysis was performed by performing an unpaired t-test with Benjamini–Hochberg FDR correction. Metabolites with P < 0.01 and fold change > 2 were considered significant. Peak annotations were performed using the METLIN (metlin.scripps.edu) metabolite databases, with a mass error of less than 15 ppm.

**Bacterial pigment extraction and thin-layer chromatography**

Bacterial pigments were extracted in a 7:2 mixture of acetone and methanol. A pellet derived from 10 ml culture was re-suspended in a minimum volume of medium/buffer, 1 ml of the solvent mixture was added, and the suspension was incubated for1 h at room temperature in the dark, vortexed, and centrifuged at 9000 × g for 10 min. For thin layer chromatography, reverse phase plates of silica gel 60 RP-18 F_245_S (Merck) were used; the mobile phase was a 60:1 mixture of dichloromethane and methanol. After running the chromatogram for 4 h, the chromatography plate was photographed using an I-phone camera. Later, the contrast settings of the image were adjusted using the Microsoft Powerpoint program to make the developed spots visible.

**UV-visible spectra of pigments**

50 μl of pigment extract as dissolved in 1 ml of PBS buffer, and 1 μl of this solution was applied to a Nanodrop One Uv-visible spectrophotometer. PBS buffer was used as blank. Measurements were performed in wavelength scan mode from 200-800 nm. The measured data was plotted using Microsoft Excel.

**References**

Blin, K., Shaw, S., Augustijn, H. E., Reitz, Z. L., Biermann, F., Alanjary, M., Fetter, A., Terlouw, B. R., Metcalf, W. W., Helfrich, E. J. N., van Wezel, G. P., Medema, M. H. and Weber, T. (2023) antiSMASH 7.0: new and improved predictions for detection, regulation, chemical structures and visualisation. *Nucleic Acids Research* 51 (W1), W46-W50.

Blin, K., Shaw, S., Steinke, K., Villebro, R., Ziemert, N., Lee, S. Y., Medema, M. H. and Weber, T. (2019) antiSMASH 5.0: updates to the secondary metabolite genome mining pipeline. *Nucleic Acids Research* 47 (W1), W81-W87.

Letunic, I. and Bork, P. (2021) Interactive Tree Of Life (iTOL) v5: an online tool for phylogenetic tree display and annotation. *Nucleic Acids Res* 49 (W1), W293-W296.
